# Harnessing Moderate-Sized Language Models for Reliable Patient Data Deidentification in Emergency Department Records: Algorithm Development, Validation, and Implementation Study

**DOI:** 10.2196/57828

**Published:** 2025-04-01

**Authors:** Océane Dorémus, Dylan Russon, Benjamin Contrand, Ariel Guerra-Adames, Marta Avalos-Fernandez, Cédric Gil-Jardiné, Emmanuel Lagarde

**Affiliations:** 1 AHeaD Team, University of Bordeaux, INSERM, BPH, U1219, 146 Rue Léo Saignat, Bordeaux, F-33000, France, 33 5 57 57 15 04; 2 SISTM Team, University of Bordeaux, INSERM, INRIA, BPH, U1219, Bordeaux, F-33000, France; 3 Department of Emergency Medicine, Bordeaux University Hospital, Bordeaux, F-33000, France

**Keywords:** de-identification, machine learning, large language model, natural language processing, electronic health records, transformers, general data protection regulation, clinical notes

## Abstract

**Background:**

The digitization of health care, facilitated by the adoption of electronic health records systems, has revolutionized data-driven medical research and patient care. While this digital transformation offers substantial benefits in health care efficiency and accessibility, it concurrently raises significant concerns over privacy and data security. Initially, the journey toward protecting patient data deidentification saw the transition from rule-based systems to more mixed approaches including machine learning for deidentifying patient data. Subsequently, the emergence of large language models has represented a further opportunity in this domain, offering unparalleled potential for enhancing the accuracy of context-sensitive deidentification. However, despite large language models offering significant potential, the deployment of the most advanced models in hospital environments is frequently hindered by data security issues and the extensive hardware resources required.

**Objective:**

The objective of our study is to design, implement, and evaluate deidentification algorithms using fine-tuned moderate-sized open-source language models, ensuring their suitability for production inference tasks on personal computers.

**Methods:**

We aimed to replace personal identifying information (PII) with generic placeholders or labeling non-PII texts as “ANONYMOUS,” ensuring privacy while preserving textual integrity. Our dataset, derived from over 425,000 clinical notes from the adult emergency department of the Bordeaux University Hospital in France, underwent independent double annotation by 2 experts to create a reference for model validation with 3000 clinical notes randomly selected. Three open-source language models of manageable size were selected for their feasibility in hospital settings: Llama 2 (Meta) 7B, Mistral 7B, and Mixtral 8×7B (Mistral AI). Fine-tuning used the quantized low-rank adaptation technique. Evaluation focused on PII-level (recall, precision, and *F*_1_-score) and clinical note-level metrics (recall and BLEU [bilingual evaluation understudy] metric), assessing deidentification effectiveness and content preservation.

**Results:**

The generative model Mistral 7B performed the highest with an overall *F*_1_-score of 0.9673 (vs 0.8750 for Llama 2 and 0.8686 for Mixtral 8×7B). At the clinical notes level, the model’s overall recall was 0.9326 (vs 0.6888 for Llama 2 and 0.6417 for Mixtral 8×7B). This rate increased to 0.9915 when Mistral 7B only deleted names. Four notes of 3000 failed to be fully pseudonymized for names: in 1 case, the nondeleted name belonged to a patient, while in the others, it belonged to medical staff. Beyond the fifth epoch, the BLEU score consistently exceeded 0.9864, indicating no significant text alteration.

**Conclusions:**

Our research underscores the significant capabilities of generative natural language processing models, with Mistral 7B standing out for its superior ability to deidentify clinical texts efficiently. Achieving notable performance metrics, Mistral 7B operates effectively without requiring high-end computational resources. These methods pave the way for a broader availability of pseudonymized clinical texts, enabling their use for research purposes and the optimization of the health care system.

## Introduction

The digitization of medical data has profoundly transformed health care, facilitating the easy and efficient sharing of patient information [1]. This digital transition, embodied by electronic health records systems, offers promising opportunities for data-driven solutions, research, and surveillance on a pan-European scale [2]. Yet, alongside the many advantages of digitization come significant concerns about the privacy and security of sensitive patient data [3]. The European General Data Protection Regulation emphasizes the necessity of stringent data protection measures, particularly for health-related information [2]. Clinical notes, which often encompass identifiable patient details, must adhere to these standards to safeguard patient confidentiality [loi informatique et liberté], before any data sharing researchers face the critical task of developing and integrating methods that mask sensitive data, guaranteeing protection against any unauthorized access [4]. Our team was recently faced with this challenge in a project aimed at classifying clinical notes from emergency services to extract the necessary information for the establishment of a trauma observatory [5].

Manual deidentification of medical records is not feasible, as it is expensive in terms of personnel resources and the time required to accomplish the task. Alternatively, multiple strategies have been implemented for the automated deidentification of medical records [6,7]. These methods evolved from systems based on explicit rules, regular expressions or dictionaries [8-16], to techniques using machine learning [17-19].

In recent years, the evolution of language models, particularly those based on transformer architectures, has reshaped the landscape of natural language processing (NLP). Transformers, introduced by Vaswani et al [20] in 2017, provided a novel approach to handling sequential data using self-attention mechanisms, thereby obviating the need for recurrent layers and significantly augmenting training efficiency. This pivotal innovation paved the way for the advent of progressively sophisticated and expansive models. Transformer-based language models of a moderate scale, particularly through customized and fine-tuned versions of the architecture BERT [21], have demonstrated high capabilities in various health care applications. These models excel in understanding and processing complex clinical texts, enabling tasks such as predicting patient outcomes and identifying medical events. For instance, a recent study highlighted the effectiveness of fine-tuned BERT models in analyzing clinical notes to predict occurrences of falls, showcasing the model’s ability to comprehend subtle nuances in medical language [22]. Additionally, BERT models offer significant benefits for tasks such as named entity recognition (NER). Those models offer notable benefits for deidentification, thanks to their capacity to discern patterns among words and phrases. They have the ability to learn from diverse text types means they can effectively tackle various pseudonymization challenges, as they can be trained to erase a wide range of identifiable details across different document types.

The burgeoning of computational resources and datasets has since kindled a shift toward the construction of massive models, embedded with trillions of parameters [23-25]. As they grew in size, their generalization aptitude and versatility witnessed substantial enhancement, optimizing tasks such as deidentification. In 2023, Liu et al [25] underscored the potential of leveraging the GPT-4’s inherent capacity for 0-shot in-context learning. A salient highlight of their methodology was its ability to maintain the original structure and meaning of the text after the removal of confidential details. While the capabilities of GPT-4 are undeniable, its application in the realm of health care presents serious ethical and legal dilemmas, primarily concerning data privacy and patient confidentiality. On the one hand, due to the vastness of the model, local hosting of GPT-4 is not feasible, therefore, data should be transmitted to external servers, in this case OpenAI’s infrastructure. On the other hand, considering the confidentiality of the weights, only locally hosted servers are regulatory compliant. Furthermore, considering that GPT-4 is a proprietary model, organizations cannot fully control or audit the underlying mechanics or data handling processes.

From a regulatory perspective, sending personal health information externally contravenes many data protection regulations, most notably the General Data Protection Regulation in Europe and the Health Insurance Portability and Accountability Act [26,27] in the United States. This raises not just data sovereignty issues but also infringes on patient rights, as they might not have explicitly consented for their data to be processed in external environments. Hence, while the technological feats of models such as GPT-4 are commendable, their real-world applications, especially in sensitive sectors such as health care, require careful consideration and possibly, significant adjustments to ensure full regulatory compliance and ethical integrity.

Generative language models significantly smaller in size (several billion parameters compared to over a trillion for GPT-4) have been recently developed and made available to the public under licenses that allow for almost unrestricted use (Llama 2 by Meta [28]) or even under open-source terms (Mistral [29]).

The objective of our study is to design, implement, and evaluate deidentification methods involving proper prompt engineering and fine-tuning of 3, open-source language models (Llama 2 7B, Mistral 7B, and Mixtral 8×7B [30]). These models were selected for their moderate size, making them suitable for deployment on personal computers for production inference tasks.

## Methods

### Study Design

We first attempted to perform the task using only prompt engineering and 0-shot inference. As we failed to achieve any significant results, we improved the selected models’ capability to deidentify clinical texts using quantized low-rank adaptation [31] fine-tuning with a dataset of instruction or response pairs. In practice, the task consists in replacing personal identifying information (PII; name, location, dates, telephone number, email, or identification numbers) with generic placeholders, represented as “[XXXXX],” or, when no PII is detected, by generating the text as “ANONYMOUS.” The ultimate goal of this procedure is to preserve text content, ensuring adherence to privacy and confidentiality requirements.

### Data Source, Datasets Allocations, and Annotation

**Figure 1.**
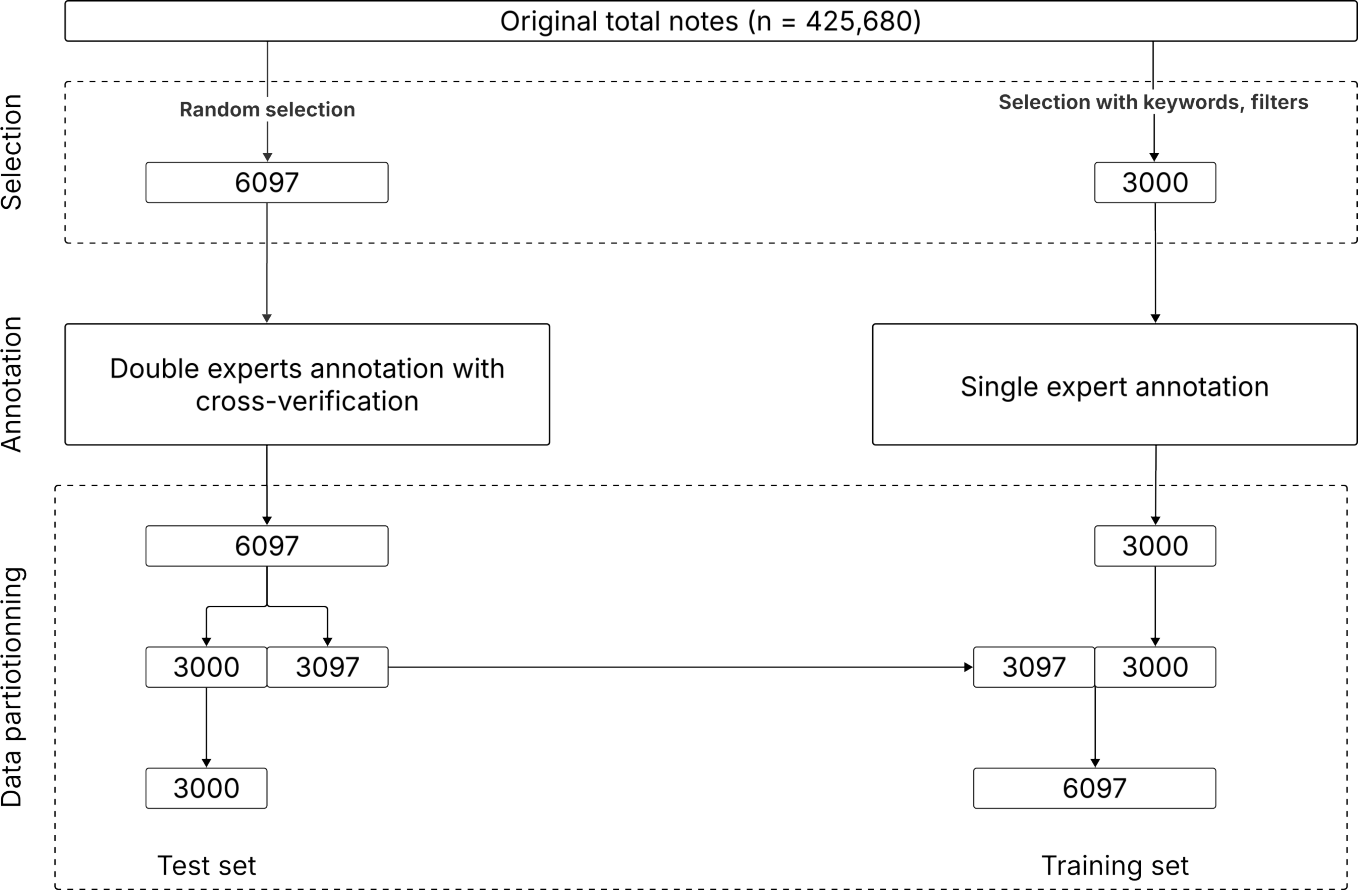
Data preparation: annotation and splitting into training and test sets.

Within the emergency department, triage is conducted by triage nurses. This process involves the collection of information on each patient, including medical history, current symptoms, vital signs, and personal details. It is these data that we have at our disposal in our study. For this investigation, we curated our dataset from a repository containing 425,680 clinical free-text notes (Multimedia Appendix 1), authored by a nurse during the initial reception and triage of individuals at the Bordeaux University Hospital’s adult emergency department over the period spanning from January 2013 to December 2022. A subset of 6097 clinical notes was randomly selected and independently annotated by 2 experts. Any arising discrepancies were adjudicated by a third expert, thus establishing a reference database. From this curated sample of 6097 clinical notes, 3000 were delineated to constitute a test dataset, upon which accuracy metrics were evaluated (Figure 1). The residual 3097 clinical notes, alongside an additional sample of 3000 clinical notes designed using filters and keywords search to encompass a broad spectrum of identifying scenarios, comprised the validation dataset.

In order to further assess whether the deidentification performances of the models varies with the type of PII, we classified identifying information within clinical notes into 6 distinct categories (Table 1). These categories were used by annotators to label such information in the test dataset. While we have taken care to remove obvious PII such as names, addresses, and identification numbers, it is important to note that deidentification cannot be considered as a strict anonymization process. For instance, in cases of rare diseases or very specific descriptions, reidentification could theoretically be possible. As every clinical history is unique, ensuring complete anonymity is unattainable. Our goal is to pseudonymize data, striking a balance between patient confidentiality and data utility for research, as removing all sensitive information will significantly diminish the data’s usefulness.

**Table 1.** Personal identifying information categories description in medical records.

Type	Code	Description
Individual names	NAME	Includes both first and last names of individuals (including patients and medical staff) or of relatives, employers, or household members of the individuals, ensuring personal identification.
Dates	DATE	Pertains to specific dates related to medical events, appointments, or personal milestones, formatted as day, month, or year.
Geographic identifiers	LOC^a^	Covers names of geographic locations such as cities, medical facilities, or addresses, facilitating location-based identification.
Phone numbers	TEL^b^	Comprises all forms of telephone numbers for direct contact, including mobile and landline numbers.
Email addresses	MAIL	Encompasses electronic mail addresses, allowing for digital communication.
Miscellaneous identifiers	OTHER	A catch-all category for unique identifiers not covered by other categories, including social security numbers, medical analysis codes, and URLs for patient images.

a LOC: location.

b TEL: telephone.

### Selected Models

We have selected 3 language models that share the following 2 characteristics: being open-source and of sufficiently small size for the production phase to be implemented on affordable PC-type systems. These are Llama 2 7B, Mistral 7B, and Mixtral. Llama 2 7B is developed by Meta. Launched in 2023, this is a 7-billion-parameter model, which is claimed to exhibit a good balance between performance and efficiency. We also selected the Mistral 7B model, introduced to the public in October 2023. It has demonstrated superior performance, either matching or surpassing that of Llama 2 13B in extensive benchmarks and showing comparable results to Llama 1 34B in specific domains such as reasoning, mathematics, and code generation. In December 2023, the Mixtral 8×7B model was released. It is described as a Sparse Mixture of Experts language model. Its key innovation lies in the routing of inference tasks through 1 selected expert out of 8, enabled by an additional routing layer. Consequently, despite its 8×7B size with respect to fine-tuning, Mixtral achieves a significant efficiency by requiring an eightfold reduction in parameters for inference task.

### Fine-Tuning and Inference

Each model was subjected to the same prompt or response pairs of clinical notes. The fine-tuning process was uniformly standardized across all 3 models, albeit with variations in batch sizes and quantization rates to accommodate our hardware constraints. The fine-tuning configuration for Mistral 7B and Llama 2 7B involved a batch size of 24 records per GPU, while Mixtral used a batch size of 20. The models were fine-tuned over 15 epochs, using the AdamW optimizer [32] with a learning rate of 5e-5 and a weight decay of 0.01. We used the quantized low-rank adaptation technique, allowing for specific adjustments in selected parts of the model, such as query, key, value, output, and gates projection modules while preserving the overall architecture integrity. The low-rank adaptation configuration included a rank setting of 32, a learning rate multiplier (alpha) set to 64, with a dropout of 0.1, and without any bias setting. Additionally, to optimize computational efficiency and minimize memory consumption, the models were quantized to 8-bit precision for both 7B models, and 4-bit precision for Mixtral. At every fine-tuning epoch, the inference was induced for each model.

The computational undertakings of this research were performed on a server running Ubuntu (version 22.04; Canonical Ltd), outfitted with 4 A100 GPUs, collectively boasting 320GB of VRAM.

### Evaluation

#### Overview

In evaluating the deidentification performance of personal data within clinical notes, our analysis is structured around 2 primary methodologies. The first methodology operates at the PII-level, enabling us to provide estimates of recall, precision, and *F*_1_-scores that are comparable with previous work in the literature. The second methodology focuses on clinical notes as the statistical unit, enabling us to assess the variation in recall performance according to the category of PII. This latter approach needs to be complemented by the measurement of a BLEU (bilingual evaluation understudy) score to assess potential modifications in the text. The assessment of the number of successful deidentifications was conducted through a comparison with the manually annotated test dataset.

#### PII-Based Metrics

This approach centers on treating each PII as an independent statistical unit. This perspective allows us to gauge the precision and recall of our deidentification efforts at the most granular level. Recall in this context is conceptualized as the proportion of PIIs accurately identified and removed from the clinical notes.


$$Recall\;PII=\frac{number\;of\;correctly\;deidentified\;PII\;per\;clinical\;notes}{total\;number\;of\;PII\;per\;clinical\;notes}$$

Precision, meanwhile, reflects the accuracy of our model in identifying and eliminating actual PIIs, distinguishing between correct identifications and false positives.


$$Precision\;PII=\frac{number\;of\;correctly\;deidentified\;PII\;per\;clinical\;notes}{total\;number\;of\;PII\;tagged}$$

The summary *F*_1_-score measure is:


$$F_{1}-score=\frac{\frac{2}{1}}{\text{precision}+\frac{1}{\text{recall}}}$$

#### Clinical Note–Based Metrics

The second approach adopts the entire clinical note as the statistical unit of analysis. Here we evaluate the success of deidentification on a document-wide scale, marking a “success” when every PII within a note has been successfully deidentified. Such a measure offers insight into the overall effectiveness of our deidentification protocols. Recall, in this instance, measures the ratio of fully deidentified notes to those containing any PII.


$$Recall=\frac{number\;of\;correctly\;de-identified\;clinical\;notes\;among\;identifying\;clinical\;notes}{total\;number\;of\;identifying\;clinical\;notes}$$

Because the clinical notes in the validation set are annotated by indicating the nature of the PII (according to the categories in Table 1), it is possible to detail the variations in recall by category. The relevance of precision is altered in this context, as it necessitates a different consideration of what constitutes a pseudonymization attempt, denoted by the presence of a pseudonymization tag. Instead, the potential alteration of content possibly induced by the deidentification process was measured using the BLEU score [33].


$$BLEU=BP\cdot exp\left(\sum w_{n}\;log\;p_{n}\right)$$

where BP is the brevity penalty, w_n_ the weight for each n-gram, and p_n_ the precision of n-grams. We set a value of 4 for the BLEU score calculation, aligning with common practice in NLP to capture up to 4-gram coherence, thereby ensuring a comprehensive evaluation of content preservation.

### Ethical Considerations

#### Overview

This study was conducted as part of the Automated Processing of Emergency Department Visit Summaries for a National Observatory project, which aims to automate the processing of emergency department visit summaries for national observation purposes.

The study received the following regulatory approvals: (1) the Ethics Committee for Research in Science and Health, validating the compliance of the protocol with current ethical requirements; and (2) the National Commission on Informatics and Liberty, under decision DR-2022-235 (authorization request 922170), allowing the processing of data for this study.

#### Confidentiality and Data Protection

The data processing was carried out exclusively on a secure local server, specially dedicated to this purpose. This server meets the current security standards, ensuring the confidentiality, integrity, and protection of the processed information. All necessary technical and organizational measures have been implemented to prevent unauthorized access to the data and to ensure strict compliance with regulatory requirements.

#### Compensation

Since this study relies solely on the analysis of pre-existing medical data and does not require direct patient involvement, no financial compensation was provided.

## Results

### Data Overview

Very few notes contained PIIs categorized as email addresses and “other.” These categories are included in the training sample due to an ad hoc selection process, which used filters to ensure representation, as half of the set was selected this way. Our examination of the test sample, which consists entirely of randomly selected clinical notes, reveals that names, places, and dates are the most prevalent types of PII. The categories of identifying data in the training and test sets are summarized in Table 2.

Regarding the length of clinical notes, they range from 8 to 3916 characters (with an average of 443, SD 289 characters) in the training set and from 3 to 2138 characters (averaging 439, SD 283 characters) in the test set. A total of 935 (31.2%) clinical notes in the test set contain at least one PII.

**Table 2.** Enhanced distribution of PII^a^ in train and tests sets.

	Train set	Test set
**Clinical notes**		
Nonanonymous medical notes, n (%)	3442 (56.5)	935 (31.2)
Randomly selected medical notes, n	3097	3000
Ad hoc selected medical notes, n	3000	—^b^
Total count, n	6097	3000
**PII categories, n**		
NAME	3016	555
LOC^c^	1801	715
TEL^d^	650	41
EMAIL	13	0
DATE	2404	607
OTHER	33	1
Total number of PII	7917	1919

a PII: personal identifying information.

b This corresponds to the absence of ad-hoc selected medical notes.

c LOC: location.

d TEL: telephone.

### Performance Using PII-Based Metrics

**Figure 2.**
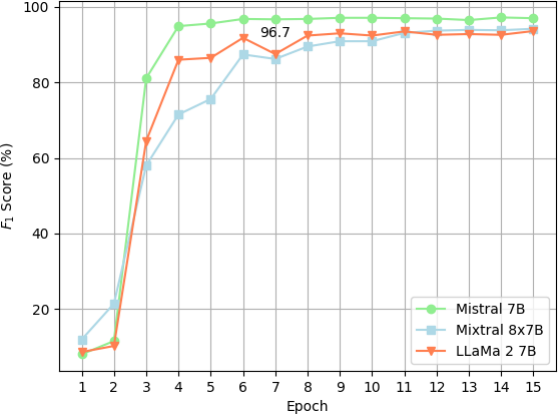
Plot of *F*_1_-score by epoch: PII as statistical unit.

Figure 2 plots the change in the *F*_1_-score over the 15 epochs of fine-tuning for the 3 respective models. The Mistral 7B model quickly reaches a performance plateau, where its *F*_1_-score stabilizes, whereas the Mixtral 8×7B and Llama 2 7B models exhibit a slower rate of improvement, with both reaching a plateau in their *F*_1_-scores around the 12th epoch.

### Recall Analysis

**Figure 3.**
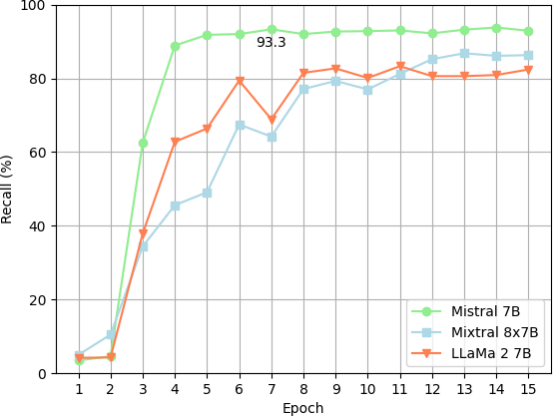
Plot of recall by epoch: clinical notes as statistical unit.

The recall estimates of the 3 models are shown in Figures3  4.

Mistral 7B and Mixtral 8×7B achieved better overall recall. The Mistral 7B and Mixtral 8×7B models demonstrated marked enhancements in their deidentification efficacy across epochs, starting from the third epoch onward. Notably, the Mistral 7B model has shown a rapid improvement in performance, achieving a performance plateau by the sixth epoch. Conversely, the Mixtral 8×7B model’s improvement trajectory was more gradual, reaching a stable performance level by the 13 epoch. The overall success rate appears not to improve beyond epoch 7 for the Mistral 7B model. Consequently, in the subsequent analysis, this epoch was selected for comparing success rates across categories.

As shown in Figure 5, Mistral 7B consistently outperformed Mixtral 8×7B and Llama 2 across all data identification categories. Despite Mixtral’s performance improving over time, it still did not surpass Mistral 7B. Using Mistral 7B, a 100% (41/41) recall was observed for phone numbers (Figure 5) and recall was lower for locations than for names.

**Figure 4.**
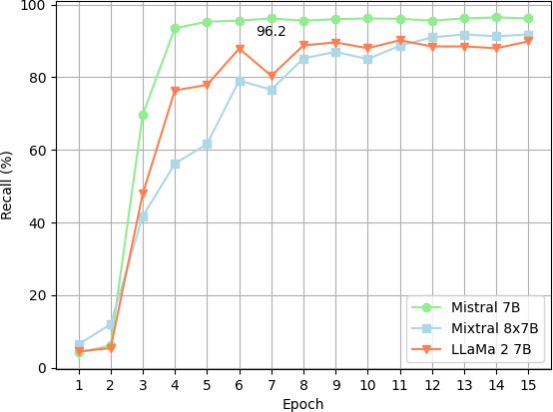
Plot of recall by epoch: PII as statistical unit. PII: personal identifying information.

**Figure 5.**
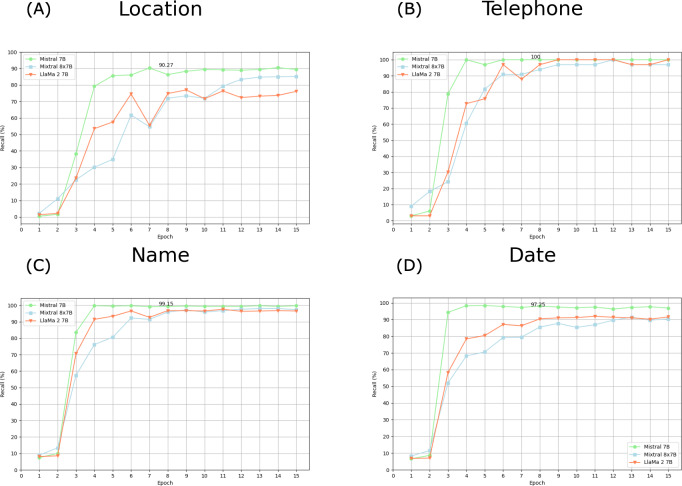
Plot of recall by epoch for PII: (A) Location, (B) Telephone, (C) Name, (D) Date. PII: personal identifying information.

### BLEU Score

**Figure 6.**
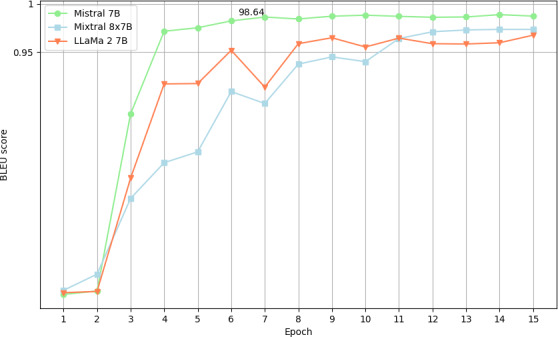
Plot of BLEU score by epoch: clinical note as statistical unit. BLEU: bilingual evaluation understudy

BLEU-4 scores were calculated to assess whether the models modified the texts at the note level. During the deidentification process, medical texts remained almost unchanged as demonstrated by a consistently high BLEU-4 score (Figure 6) beyond epoch 5.

### Results Summary at Epoch 7

The Table 3 below presents a summary of performance metrics achieved by our models at epoch 7.

The results demonstrate that the Mistral 7B model outperforms both the Mixtral 8×7B and Llama 2 7B with a *F*_1_-score of 0.9673. When using clinical note as the statistical unit, the recall is also much higher (0.9326) for Mistral 7B than Llama 2 and Mixtral 8×7B models.

**Table 3.** Fine-tuned models performance at epoch 7.

Model	Clinical notes	Personal identifying information		
	Recall	Precision	Recall	*F*_1_-score
Mistral 7B	0.9326	0.9721	0.9625	0.9673
Llama 2 7B	0.6888	0.9596	0.8041	0.875
Mixtral 8×7B	0.6417	0.9852	0.7655	0.8616

### Error Analysis

In epoch 7 of the Mistral 7B model, a total of 63 clinical notes were not properly pseudonymized, as detailed in Table 4. Among these, location (LOC) errors were the most frequent, with 44 instances. Deleting geographical and institutional identifiers then remains a significant challenge (with a recall of 86.1%). Specifically, 31 notes still included names of health or social service facilities, while 12 notes still included names of cities. Conversely, errors involving names (NAME) were significantly fewer, with only 4 instances, including 1 patient name and 3 doctors’ names, resulting in a high recall of 99.8% for this category. Date-related errors (DATE) were observed in 14 notes (with a recall of 97.8%).

The test dataset, comprising 3000 clinical notes, underwent a post hoc examination to identify any inaccuracies resulting from manual annotations that would have been detected by all 15 versions of our 3 finely-tuned models, spanning epochs 1 to 15. Through this process, we were able to pinpoint 65 notes in which the model detected personally identifiable information through the medical histories that were categorized as anonymous (ie, without identifying data, 2066 clinical notes), in which the model detected personally identifying information that had been overlooked by human annotators.

**Table 4.** Summary of deidentification errors at epoch 7.

Errors	Count
Total	63
Returned ANONYMOUS	29
Annotation error	34
**Errors in personal identifying information categories**	
NAME	4
LOC^a^	44
DATE	14
OTHER	1

a LOC: location.

We observed that the models outperformed human annotation in 9 clinical records from the test set. Specifically, in these 9 records, 5 locations (LOC), 3 names (NAMES), and 1 date (DATE) were omitted during manual annotation. The remaining 53 records present annotation errors from the models. Therefore, the total number of actual personally identifiable information (PII) amounts to 1928, contrary to the 1919 initially identified by our experts.

Subsequently, corrections were made to the test dataset based on these findings, and main outcomes were recomputed in an additional sensitive analysis. The metric measurements after accounting for these modifications are only slightly altered from the original results (see Multimedia Appendix 2 for the details).

## Discussion

### Principal Findings

In this study, we assessed the performance of 3 generative NLP models in the deidentification of clinical text documents. The generative model Mistral 7B demonstrated the highest performance with an overall *F*_1_-score of 0.9673. At the clinical notes level, the same model achieved an overall recall of 0.9326, with this rate increasing to 0.9915 for the deletion of names. The evaluation was based on a test dataset of 3000 clinical notes, among which only 4 notes failed to be fully deidentified for names; in one case, the identifying name was that of a patient. As the method relies on the use of generative models, we also measured potential text alterations generated by the process. Beyond the fifth epoch, the BLEU score consistently exceeded 0.9864.

### Strengths

Our work distinguishes itself from the existing scientific literature by using a method that does not rely on NER and uses moderate-sized models. Instead, the use of generative large language models allows for the production of text that is pseudonymized by removing PII components. This is the reason why we added metrics that use clinical notes as the statistical unit. This led us to use the BLEU metric to assess potential text alterations. Another consequence of this method is that no hyperparameters are set which made it possible to avoid the use of separate test and validation dataset partitions. The size of our training and test samples, independently annotated by 2 experts, constitutes a significant strength in our study. To our knowledge, no other study has used a test sample of such size (3000 notes). Yet, it is crucial to have the means to detect rare errors if the ultimate goal is to develop a system that guarantees the pseudonymization of clinical texts. We deliberately limited our model selection to those whose implementation does not require powerful servers and can be executed on personal computers equipped with a consumer-grade graphics card. The largest model is Mixtral 8×7B, which has approximately 8 times more parameters than the other 2 models. Mixtral 8×7B shares the same architecture as Mistral 7B, with the distinction that each layer consists of 8 feed-forward blocks. Although training it requires significant memory capacity, this is not the case during the inference phase, during which only 2 of the feed-forward blocks are used, selected by a network acting as a router.

### Limitations

#### Annotation Process Inaccuracies

##### Overview

During the annotation process, we observed some inaccuracies. To assess the impact of these inaccuracies on our metrics, we conducted a post hoc analysis, taking into account corrections made by the model. Although this analysis revealed few variations, it is important to note that some errors may still remain in the text set, undetected by the model. These undetected errors could potentially affect the overall performance of the model.

##### Model Choice

We opted for a fine-tuned large language model–based approach over a dedicated NER model due to pragmatic considerations. Our hypothesis was that a targeted human annotation process, with expert annotators pinpointing PII within texts, would be more effective than a broad NER annotation effort, given the same time investment. Focusing on essential PII elements helps us minimize the ambiguities that broader NER annotations often entail. This focus leads to improved precision and recall rates during the training phase. Furthermore, this approach is in line with the Automated Processing of Emergency Department Visit Summaries for a National Observatory project’s objectives, which prioritize the accurate removal of PII from unstructured medical texts.

The default choice for identification tasks is usually a bidirectional transformer, starting from the hypothesis that the relationship of a word with its context before and after that word allows for better comprehension of the role of those words and therefore should be more suited for NER tasks. However, this hypothesis no longer holds when dealing with generative models. Since the goal here is to generate redacted text, the provided prompt has access to the entire corrected phrase. Consequently, relative to a given word, implications cannot be considered unidirectional.

### Model Sharing Constraints

#### Overview

Another significant limitation is that our model was fine-tuned using nonanonymous clinical texts, which prevents us from sharing the model’s weights with the community. Sharing the model’s weights could potentially allow for the extraction of the original training data. This limitation restricts the model’s reproducibility and its broader applicability across different research settings and medical domains.

#### Demographic and Textual Bias

The processed data are in free-text format, written by health care staff, which introduces significant variability. This variability is not only present between different services within the same health facility but also across various centers. Factors such as the content of clinical notes, the medical abbreviations used, writing styles, and the level of detail in documentation can differ greatly from one source to another. Such differences could potentially impact the performance of our models, making it essential to test and adapt our approach to data from diverse sources.

### Comparison With Prior Work

Comparing the performance of our models with those documented in the literature presents challenges because our models are specifically fine-tuned to pseudonymize French-language clinical notes. Consequently, it is not feasible to apply them to the English-language databases traditionally used for benchmarking, such as i2b2 (i2b2 TranSMART Foundation) [34], MIMIC II (PhysioNet) [35], and MIMIC III (PhysioNet) [36].

In addition to these differences in benchmarking context, there are also divergences in the methodologies used for deidentification. Historically, deidentification of medical records has evolved from rule-based systems, which rely on predefined rules, regular expressions, and dictionaries, to more sophisticated machine learning approaches. Rule-based methods, while easy to implement and interpret, often fall short in handling the variability and unpredictability inherent in unstructured clinical texts. On the other hand, machine learning-based approaches offer more flexibility and adaptability, particularly when dealing with large and diverse datasets. These models can learn patterns directly from the data, making them more effective in identifying PIIs that deviate from standard formats. However, their effectiveness is heavily dependent on the quality and quantity of annotated data available for training. Moreover, machine learning models typically require significant computational resources and expertise in model tuning, which can be a barrier to adoption, particularly in resource-constrained settings.

Our proposed model leverages these advanced machine learning techniques, specifically fine-tuned for the French language. This focus allows our model to effectively capture and manage the linguistic intricacies specific to French clinical notes, such as frequent abbreviations and unstructured text entries, which are common in emergency department settings.

Additionally, our results demonstrate that while our model performs comparably to those trained on English-language corpora, certain challenges persist, particularly in the detection of location-based PIIs. This is likely due to the complexity introduced by variations in PII forms, such as acronyms and abbreviations, as well as the presence of typing errors, which are less predictable and harder to model.

Therefore, to compare performance metrics accurately, it is necessary to assess the complexity of clinical texts from these databases against those used in our study. In the Multimedia Appendix 1, we include examples of clinical notes from our dataset to demonstrate that PIIs can appear randomly within the text, in an unstructured manner, and that these PIIs, along with the rest of the text, often include numerous abbreviations. This tendency toward abbreviation is explained by the unique demands of emergency department settings, where nurses are required to perform efficient, real-time data entry into the hospital’s information system. As a result, our dataset more closely aligns with MIMIC II, which features unstructured clinical notes made by nurses, as opposed to i2b2, where each type of information is distinctly separated, preventing the amalgamation of multiple PIIs within single sentences.

As shown in Multimedia Appendix 3 [37-43], our results (overall *F*_1_-score of 0.9673) are on par with previous studies on English clinical text corpus that used an algorithm including models using self-attention [17,24,36,44]. The Multimedia Appendix 4 [37,38,43] summarizes study results that examined recall variations according to PII categories. These figures consistently show that the relative weakness of these algorithms, ours included, lies in a small number of errors concerning locations. Our dataset presents additional challenges for PII identification due to the presence of multiple variations of PII, including acronyms, abbreviations, and typing errors. Specifically, of the 44 notes with failed identification, 15 involved abbreviations or acronyms, and 2 contained typing errors.

### Future Work

We aim to enhance the detection capabilities of PII in our medical notes by fine-tuning our model with newly annotated data. To achieve this, we plan to generate artificial clinical notes using commercially available application programming interfaces, such as GPT-4. These large language models, much more powerful than ours, can produce realistic notes containing PII and annotations, which will facilitate the training process and increase data diversity.

By generating a substantial volume of these artificial data, we can ensure equitable representation of different PII categories and evaluate 2 key aspects: identifying the optimal amount of clinical notes needed to achieve the highest possible accuracy and recall, and comparing the effectiveness of models fine-tuned with real data versus those fine-tuned with artificially generated data.

Using this newly developed model based on artificial data, we aim to make it available as an open-source resource, benefiting the broader community. Additionally, this foundation will enable us to create a multilingual model capable of processing both English and French clinical notes. This multilingual model will allow us to perform performance comparisons against literature benchmark datasets such as i2b2 and MIMIC. The performance of these refined models will be evaluated using our corrected test set, along with newly annotated data from various emergency services.

This study is currently focused on data from an emergency department in France. In the subsequent phases, our goal is to extend this methodology to other services across France, with the ambition of creating a national French observatory on trauma. However, it is important to consider the potential for demographic biases in our model’s performance.

By diversifying data sources, we aim to enhance the model’s generalizability. If biases are identified in this process, we plan to retrain the model, either by using a specific portion of data from each service or by integrating synthetic data to mitigate these biases.

We intend to extend our methodology to other types of sensitive documents, such as medico-legal records, to evaluate the generalizability and effectiveness of our approach in protecting personal information across various domains.

We are also considering integrating explainability methods, similar to those used by Arnaud et al [45], to enhance the transparency of our model in PII detection. These techniques, based on transformer models and interpretability approaches such as LIME [46], which have already proven effective on triage note data similar to ours, could strengthen user trust and facilitate the adoption of our technologies in clinical settings.

Through this comprehensive approach, we aim to enhance the value and applicability of our models, contributing to the development of privacy-preserving technologies in the health care domain and strengthening the security of patients’ sensitive information.

### Ethical Considerations and Practical Implementations

The use of small to moderate-sized models is a key consideration in our approach. These models are generally capable of running on GPUs with at least 16 GB of VRAM, making them suitable for use on personal computers or within local infrastructures. This is particularly advantageous for institutions with limited resources, as it allows them to manage data privately and securely without relying on extensive external infrastructure. However, while local deployment ensures better control over sensitive data, it can also be time-consuming and may introduce challenges related to the interoperability of different systems.

One of the main challenges of this pipeline is its implementation across all participating emergency services, given that not all institutions may be equipped to efficiently manage these new procedures. The rationale behind implementing this process is rooted in a data-sharing initiative aimed at establishing a national observatory, which necessitates enhanced protection for the information being used.

At this stage, centralizing the data in a dedicated center with the necessary computational resources remains the simplest solution. This would allow for secure, controlled, and efficient management of patient data. Alternatively, the process could be implemented directly within health data warehouses, enabling these facilities to store and apply the deidentification process locally. Regardless of the approach, it is imperative that the use of this pipeline on health data is conducted within a legally and digitally controlled framework, authorized by the relevant authorities.

Given the potential risks of data reidentification, especially when dealing with unique clinical histories, we emphasize that pseudonymization alone is insufficient and should be accompanied by additional protection and security measures to prevent unauthorized access to sensitive data.

### Conclusion

Our research underscores the significant capabilities of generative NLP models, with Mistral 7B standing out for its superior ability to deidentify clinical texts efficiently. Achieving notable performance metrics, Mistral 7B operates effectively without requiring high-end computational resources. These methods pave the way for a broader availability of pseudonymized clinical texts, enabling their use for research purposes and the optimization of the health care system.

## Supplementary material

10.2196/57828 Multimedia Appendix 1 Examples of French nursing notes.

10.2196/57828 Multimedia Appendix 2 Analysis of performance evaluation on corrected test set.

10.2196/57828 Multimedia Appendix 3 Comparative table of statistical results from previous studies.

10.2196/57828 Multimedia Appendix 4 Comparative table of recall across PII categories from previous studies. PII: personal identifying information.

## Abbreviations

BLEU: bilingual evaluation understudy
NER: named entity recognition
NLP: natural language processing
PII: personal identifying information
