# Supplementary material for: Harnessing Moderate-Sized Language Models for Reliable Patient Data Deidentification in Emergency Department Records: Algorithm Development, Validation, and Implementation Study
Source: JMIR AI. 2025 Apr 1;4:e57828. doi: 10.2196/57828 (PMC12223680; doi:10.2196/57828)
Supplement: Multimedia Appendix 1 [file ai-v4-e57828-s001.docx]

In the provided document, the upper section presents an original clinical text as it was written by a nurse or hospital staff. The section below displays the same note after correction, rendered in proper French.

For this first example (Textbox 1), identifying data have been pseudonymized. The location (LOC) of the original note has been replaced with 'Clinique du Louvre'.

**Textbox S1**. Example of French nursing notes.

| **Original note** : tft de la cl du louvre pour pec d une fracture du col femoral droit patient sous plavix transféré du louvre pour orthopédiste pour prise en charge d'une fracture du col du fémur  **Manual transcription without abbreviations** : Transfert de la clinique du Louvre pour prise en charge dune fracture du col femoral droit patient sous plavix transféré du Louvre pour orthopédiste pour prise en charge d'une fracture du col du fémur |
| --- |

For this second example (Textbox 2) we change the date, the name of the Doctor and the Location.

**Textbox S2.** Example of French nursing notes.

| **Original note** : chutes à repet trauma lombaires sans deficit plaie 2 pieds car a marchè sur debris verre ( a rv pour irm cerebrale à cl du louvre )- a resulttas bacterio aurait inf urinaire pas encore ttè (sa fille a resultats en salle d'attente) 01/01/2000 22:22 - docteur Dupond, interne retrouvé au sol dans les toilettes à 17h, vu pour la derniere fois à 10h par la femme de ménage. contexte d'infection urinaire, avec ecbu positif à e.coli, sensible à la rocéphine (ecbu dans le dossier). arrive avec 39 de température.  **Manual transcription without abbreviations** : Chutes à répétition, traumatisme lombaires sans déficit, plaie des 2 pieds, car a marché sur des débris verre (à rendez-vous pour irm cérébrale à clinique du Louvre)- A des résultats bacterio, elle aurait infection urinaire pas encore traitée (sa fille à les résultats en salle d'attente) 01/01/2000 22:22 - docteur Dupond, interne retrouvé au sol dans les toilettes à 17h, vu pour la dernière fois à 10h par la femme de ménage. Contexte d'infection urinaire, avec ecbu positif à e.coli, sensible à la rocéphine (ecbu dans le dossier). Arrive avec 39 de température. |
| --- |
