# Supplementary material for: Harnessing Moderate-Sized Language Models for Reliable Patient Data Deidentification in Emergency Department Records: Algorithm Development, Validation, and Implementation Study
Source: JMIR AI. 2025 Apr 1;4:e57828. doi: 10.2196/57828 (PMC12223680; doi:10.2196/57828)
Supplement: Multimedia Appendix 2 [file ai-v4-e57828-s002.docx]

In the corrected data, there are 2056 non-anonymous anamneses (compared to 2066 in the original test set) and 944 anonymous anamneses (compared to 935 in the original test set), indicating that 9 anamneses were misannotated by our experts.

### Recall, precision and F1 statistics

The Table 1 presents a comparative analysis of model performance, measured by recall, precision, and F1 score, at epoch 7 on both the original and post-hoc test sets. Bold values indicate improvements in the corrected versions of the Mistral 7B, Mixtral 8x7B, and LLaMa2 7B models, highlighting the effectiveness of fine-tuning.

**Table S1.** Comparative analysis of fine-tuned model performance at epoch 7 for original test set and post-hoc test set.

| Model | PII | Clinical Notes | | |
| --- | --- | --- | --- | --- |
|  | Recall | Precision | Recall | F1 |
| Mistral 7B | 0.9326 | 0.9721 | 0.9625 | 0.9673 |
| Mistral 7B^p^ | 0.9465 | 0.9732 | 0.9630 | 0.9681 |
| LLaMa2 7B | 0.6888 | 0.9596 | 0.8041 | 0.8750 |
| LLaMa2 7B^p^ | 0.7932 | 0.9583 | 0.8026 | 0.8736 |
| Mixtral 8x7B | 0.6417 | 0.9852 | 0.7655 | 0.8616 |
| Mixtral 8x7B^p^ | 0.7620 | 0.9819 | 0.7625 | 0.8584 |

^P :^ Post-hos test set

The corrected version of Mistral 7B shows improvement in all metrics, especially precision, which increased to 0.9588. Mixtral 8x7B and LLaMa2 7B corrected models also exhibit slight enhancements, with notable increases in precision and F1 score, demonstrating the positive impact of model corrections.

### Recall for PPI categories

The Table 2 provides a comparative analysis of the recall performance for different PPI categories (NAME, TEL, DATE, and LOC) evaluated at epoch 7 across both the original and post-hoc test sets for the Mistral 7B, Mixtral 8x7B, and LLaMa2 7B models, along with their corrected versions.

**Table S2.** Comparative analysis of fine-tuned model performance at epoch 7 for original test set and post-hoc test set. Recall PII.

| Model | NAME | TEL | DATE | LOC |
| --- | --- | --- | --- | --- |
| Mistral 7B | 0.9914 | 1.0 | 0.9725 | 0.9026 |
| Mistral 7B^p^ | 0.9914 | 1.0 | 0.9744 | 0.9037 |
| LLaMa2 7B | 0.9276 | 0.8787 | 0.8627 | 0.5553 |
| LLaMa2 7B^p^ | 0.9255 | 0.8787 | 0.8624 | 0.5557 |
| Mixtral 8x7B | 0.9148 | 0.909 | 0.7941 | 0.5464 |
| Mixtral 8x7B^p^ | 0.9106 | 0.909 | 0.7937 | 0.5426 |

^P :^ Post-hos test set

The results do not show significant differences in recall for the various PPI categories between the original and corrected versions of the test set. The recall rates across NAME, TEL, DATE, and LOC categories remain relatively stable, indicating that the corrections made to the models have not drastically altered their ability to correctly identify these PPI categories.
