# Supplementary material for: Harnessing Moderate-Sized Language Models for Reliable Patient Data Deidentification in Emergency Department Records: Algorithm Development, Validation, and Implementation Study
Source: JMIR AI. 2025 Apr 1;4:e57828. doi: 10.2196/57828 (PMC12223680; doi:10.2196/57828)
Supplement: Multimedia Appendix 3 [file ai-v4-e57828-s003.docx]

**Table S1.** Comparison with previous work.

| Authors | Corpus (N) | Language | Precision | Recall | F1 | Notes |
| --- | --- | --- | --- | --- | --- | --- |
| Grouin et al. (2013) [1] | Cardiology set (62) | FR | 94.8^b^ | 89.4^b^ | 92.1^b^ | Medina-CFR |
|  |  |  | 89.1^b^ | 86.5^b^ | 87.8^b^ | Medina-RB |
|  | Foetopathology set (10) |  | 75.4^b^ | 58.5^b^ | 65.9^b^ | Medina-CFR |
|  |  |  | 72.0^b^ | 72.6^b^ | 72.3^b^ | Medina-RB |
| Chazar et al. (2014) [2] | French discharge letters (508) | FR | 79.6 | 98.1 | 87.9 | Pattern matching. Use a list of authorized words |
| Catelli et al. (2020) [3] | SIRM (50) | IT | - | - | 85.61 | Bert-base (IT) cased |
|  |  | IT | - | - | 94.49 | mBERT Cased |
|  |  | IT | - | - | 83.17 | Bi-LSTM-CRF : BPEemb (IT) + Fair (IT) |
|  |  | EN\|IT | - | - | 86.19 | Bi-LSTM-CRF : Multi-BPEemb + Fair multi fast |
| Berg et al. (2020) [4] | neurology clinical unit (-) | SW | 96.07c. | 92.82^c^ | 94.4^c^ | token binary evaluation optimized for F1 model |
| Ahmed et al. (2020) [5] | i2b2 (1304) | EN | 98.74 | 95.85 | 97.28 | GRU |
|  |  |  | 99.01 | 95.12 | 97.08 | GRU-GRU |
|  |  |  | 98.74 | 95.27 | 96.98 | LSTM-GRU |
|  |  |  | 98.03 | 98.41 | 98.22 | Self-attention |
|  | MIMIC-II (486) | EN | 81.82 | 66.23 | 73.21 | GRU |
|  |  |  | 80.00 | 71.00 | 75.23 | GRU-GRU |
|  |  |  | 85.14 | 68.18 | 75.72 | LSTM-GRU |
|  |  |  | 89.20 | 82.90 | 85.90 | Self-attention |
|  | MIMIC-III (891) | EN | 99.94 | 100 | 99.97 | GRU |
|  |  |  | 99.93 | 99.99 | 99.96 | GRU-GRU |
|  |  |  | 99.94 | 99.99 | 99.96 | LSTM-GRU |
|  |  |  | 99.95 | 98.78 | 99.36 | Self-attention |
| Syed et al. (2022) [6] | i2b2 (-) | EN | 94.89 | 95.96 | 93.84 | Input Embeddings+Bi-LSTM+CRF |
|  | CoNLL-2003 (-) |  | 92.99 | 93.50 | 93.25 |  |
|  | internal corpus (100) |  | 93.86 | 93.37 | 94.31 | without mixed domain pre-training |
|  |  |  | 96.23 | 94.51 | 95.36 | with mixed domain pre-training |
| Meaney et al.(2022) [7] | 2014-i2b2 (486) | EN | 96.69 | 96.81 | 96.75 | Roberta-Large |
|  |  |  | 96.62 | 96.27 | 96.44 | Albert-XXLarge fine-tuned |
|  |  |  | 95.10 | 95.33 | 95.22 | Roberta-Base fine-tuned |
|  |  |  | 95.53 | 95.34 | 95.43 | Bert-Large fine-tuned |
|  |  |  | 93.87 | 93.85 | 93.86 | Albert-Base fine-tuned |
|  |  |  | 93.80 | 94.40 | 94.10 | Bert-Base fine-tuned |
| Tchouka et al. (2022) [8] | HNFC (375) | FR. | 94.6a | 94.9a | 94.7a | NER hybrid system |
| Liu Z. et al. (2023) [9] | 2014-i2b2 (50) | EN | - | - | - | Accuracy 0.99 Explicit prompt GPT-4 |
|  |  |  | - | - | - | Accuracy 0.929 Explicit prompt ChatGPT |
| Liu L. et al. (2023) [10] | 2014-i2b2 (-) | EN | 98.92a,c | 97.66^a,c^ | 98.29^a,c^ | BiLSTM-CRF (RoBERTA). |
|  | CardiacAI (40) |  | 95.19^a,c^ | 93.47^a,c^ | 94.32^a,c^ | BiLSTM-CRF (RoBERTA). |
|  | CardiacAI (60) |  | 94.87^a,c^ | 95.26^a,c^ | 95.07^a,c^ | BiLSTM-CRF (RoBERTA). |
| Liu J. et al. (2023) [11] | OpenDeid (700) | EN | 95.58^a,b^ | 92.42^a,b^ | 93.97^a,b^ | fine-tuned BioBERT |
|  |  |  | 95.82^a,b^ | 91.98^a,b^ | 93.86^a,b^ | fine-tuned Clinical BioBERT |
|  |  |  | 95.87^a,b^ | 92.22^a,b^ | 94.01^a,b^ | fine-tuned Discharge Summary BioBERT |
|  |  |  | 97.84^a,b^ | 95.92^a,b^ | 96.87^a,b^ | fine-tuned Discharge Summary BioBERT + cascading rules |
|  |  |  | 95.59^a,b^ | 89.35^a,b^ | 92.37^a^ | LSTM GloVe+PMC+word2vec-OpenDeID corpus word embeddings. |
| Our Model | French nursing notes (3000) | FR | 97.32 | 96.30 | 96.73 | fine-tuned Mistral 7B model with LoRA |

1. Grouin C, Zweigenbaum P. Automatic de-identification of French clinical records: comparison of rule-based and machine-learning approaches. Stud Health Technol Inform. 2013;192:476-480. [Medline: 23920600]

2. Chazard E, Mouret C, Ficheur G, Schaffar A, Beuscart JB, Beuscart R. Proposal and evaluation of FASDIM, a Fast and Simple De-Identification Method for unstructured free-text clinical records. Int J Med Inform. Apr 2014;83(4):303-312. [doi: 10.1016/j.ijmedinf.2013.11.005] [Medline: 24370391]

3. Catelli R, Gargiulo F, Casola V, De Pietro G, Fujita H, Esposito M. Crosslingual named entity recognition for clinical de-identification applied to a COVID-19 Italian data set. Appl Soft Comput. Dec 2020;97:106779. [doi: 10.1016/j.asoc.2020.106779] [Medline: 33052197]

4. Berg H, Henriksson A, Dalianis H. The impact of de-identification on downstream named entity recognition in clinical text. Presented at: Proceedings of the 11th International Workshop on Health Text Mining and Information Analysis. Nov 20, 2020:Association for Computational Linguistics. 1-11; Online. URL: <https://www.aclweb.org/anthology/2020.louhi-1>[doi: 10.18653/v1/2020.louhi-1.1]

5. Ahmed T, Aziz MMA, Mohammed N. De-identification of electronic health record using neural network. Sci Rep. Oct 29, 2020;10(1):18600. [doi: 10.1038/s41598-020-75544-1] [Medline: 33122735]

6. Syed M, Sexton K, Greer M, et al. DeIDNER Model: a neural network named entity recognition model for use in the de-identification of clinical notes. Biomed Eng Syst Technol Int Jt Conf BIOSTEC Revis Sel Pap. Feb 2022;5:640-647. [doi: 10.5220/0010884500003123] [Medline: 35386186]

7. Meaney C, Hakimpour W, Kalia S, Moineddin R. A comparative evaluation of transformer models for de-identification of clinical text data. arXiv. Preprint posted online on Mar 25, 2022. [doi: 10.48550/arXiv.2204.07056]

8. Tchouka Y, Couchot JF, Coulmeau M, Laiymani D, Rahmani A. De-identification of french unstructured clinical notes for machine learning tasks. arXiv. Preprint posted online on Oct 6, 2023. URL: <https://hal.science/hal-03720808> [doi: 10.48550/arXiv.2209.09631]

9. Liu Z, Huang Y, Yu X, Zhang L, Wu Z, Cao C, et al. DeID-GPT: zero-shot medical text de-identification by GPT-4. arXiv. Preprint posted online on Dec 21, 2023. [doi: 10.48550/arXiv.2303.11032]

10. Liu L, Perez-Concha O, Nguyen A, et al. Web-based application based on human-in-the-loop deep learning for deidentifying free-text data in electronic medical records: development and usability study. Interact J Med Res. Aug 25, 2023;12:e46322. [doi: 10.2196/46322] [Medline: 37624624]

11. Liu J, Gupta S, Chen A, et al. OpenDeID pipeline for unstructured electronic health record text notes based on rules and transformers: deidentification algorithm development and validation study. J Med Internet Res. Dec 6, 2023;25:e48145. [doi: 10.2196/48145] [Medline: 38055317]
