# Supplementary material for: Harnessing Moderate-Sized Language Models for Reliable Patient Data Deidentification in Emergency Department Records: Algorithm Development, Validation, and Implementation Study
Source: JMIR AI. 2025 Apr 1;4:e57828. doi: 10.2196/57828 (PMC12223680; doi:10.2196/57828)
Supplement: Multimedia Appendix 4 [file ai-v4-e57828-s004.docx]

**Table S1.** Comparison of recall for PII with previous work.

| Authors | Method | L. | NAME | | DATE | | LOC | | TEL | | Info |
| --- | --- | --- | --- | --- | --- | --- | --- | --- | --- | --- | --- |
|  |  |  | R (%) | N | R (%) | N | R (%) | N | R (%) | N |  |
| Grouin et al [1] | MEDINA-RB | FR | 90.7 –92.7 | 314^s^ | 87.1 | 238^s^ | 12.5-100 | 81^s^ | 100 | 8 | NAME : Last Name (205) and First Name (109)  LOC : HOSPITAL (43), TOWN(22), ZIP(8), ADDRESS(8) |
|  | MEDINA-CRF | FR | 88.3-89,0 |  | 94.6 |  | 12.5-75.0 |  | 75.0 |  |  |
| Tchouka et al [2] | FlauBERT-MEDINA | EN | 99.8 | - | 86.7 | - | 57.3-95.1 | - | 97.9 | - | LOC : ORGANIZATION + LOCATION |
| L Liu et al [3] | BiLSTM-CRF (RoBERTA) | EN | 95.83 | 528 | 96.92 | 65 | 50.0 | 6 | 85.71 | 35 | Dataset Cardaic AI (N = 600) |
| Our work | Mistral-7B + qLoRA | FR | 99.14 | 555 | 97.25 | 607 | 90.26 | 715 | 100 | 100 |  |

S : sum of entities

1. Grouin C, Zweigenbaum P. Automatic de-identification of French clinical records: comparison of rule-based and machine-learning approaches. Stud Health Technol Inform. 2013;192:476-480. [Medline: 23920600]

2. Tchouka Y, Couchot JF, Coulmeau M, Laiymani D, Rahmani A. De-identification of french unstructured clinical notes for machine learning tasks. arXiv. Preprint posted online on Oct 6, 2023. URL: <https://hal.science/hal-03720808> [doi: 10.48550/arXiv.2209.09631]

3. Liu L, Perez-Concha O, Nguyen A, et al. Web-based application based on human-in-the-loop deep learning for deidentifying free-text data in electronic medical records: development and usability study. Interact J Med Res. Aug 25, 2023;12:e46322. [doi: 10.2196/46322] [Medline: 37624624]
